# Immunogenicity of a lambda phage-based anti-cancer vaccine targeting HAAH

**DOI:** 10.1186/2051-1426-1-S1-P210

**Published:** 2013-11-07

**Authors:** Steven Fuller, Solomon Stewart, Michael Lebowitz, Kanam Malhotra, Mark Semenuk, Biswajit Biswas, Hossein Ghanbari

**Affiliations:** 1 Panacea Pharmaceuticals, Inc., Gaithersburg, MD, USA

## 

We have designed, developed and produced a lambda-phage based anti-cancer vaccine (nano-particle) targeting human aspartyl (asparaginyl) β-hydroxylase (HAAH). This follows accumulated evidence that HAAH meets requirements of a good target for anti-cancer immunotherapy. The protein is over-expressed on the surface of cancer cells and plays a central role in cancer etiology that effects cancer cell growth, motility and invasiveness. Over-expression of HAAH in transfected normal cells is sufficient to induce cellular transformation, and suppression of HAAH expression (siRNA) or neutralized activity (mAb) returns cancer cells to a normal phenotype. Moreover, tumor growth in xenograft models of human liver and lung cancer is significantly (>80%) inhibited by administration of anti-HAAH monoclonal antibodies. Therefore, it is expected that a patient polyclonal antibody response against HAAH should result in a significant therapeutic effect. HAAH is an embryonic protein and as such is a self antigen. Moreover, it has been observed that the HAAH gene is well conserved and mouse AAH has very high homology in the N-terminal portion of HAAH and complete homology in the mid and C-terminal portion. Historically, recombinant HAAH protein administered with adjuvants has not proven to be very immunogenic in mice. Here we have used immunocompetent mice to test immunogenicity of three phage-based vaccine candidates, encompassing the N-terminal, mid and C-terminal portions of the HAAH extracellular domain. All three entities display highly significant, dose-dependent immunogenicity. Animals were injected with 5x10^7^-5x10^9^ pfus on days 0, 7 and 14. Animals were bled on day 21 and immunogenicity was screened using recombinant HAAH in an ELISA format. Cell-based ELISAs using liver (FOCUS) and lung (H460) cancer cell lines as well as FACS analysis on these lines were performed. The immunized mice sera had clear anti-HAAH (or anti-cancer cell) activity in all tests. Immunogenicity was dose and construct dependent. This work demonstrates that a nano-particle, phage-based vaccine can break immune tolerance to the native HAAH protein and elicit a specific antibody response; indicating that such vaccines may have significant therapeutic value. Indeed preliminary data from an ongoing animal study testing this vaccine in a mouse tumor model has demonstrated a quick and very significant anti-tumor activity, slowing the growth of subcutaneously implanted mouse liver cancer tumors. Thus, this strategy of expressing portions of the HAAH protein on the surface of lambda-phage has resulted in overcoming tolerance to self antigen and promises to be an effective anti-cancer vaccine.

